# Difficult iatrogenic bile duct injuries following different types of upper abdominal surgery: report of three cases and review of literature

**DOI:** 10.1186/s12893-019-0619-0

**Published:** 2019-11-06

**Authors:** Jerzy Lubikowski, Bernard Piotuch, Anna Stadnik, Marta Przedniczek, Piotr Remiszewski, Piotr Milkiewicz, Michael A. Silva, Maciej Wojcicki

**Affiliations:** 1 Department of General and Oncological Surgery, Pomeranian Medical University, Szczecin, Poland; 2 Division of Hepatobiliary Surgery and Liver Transplantation, Department of Surgery, M. Curie Hospital, Szczecin, Poland; 3 Department of Surgery, Ministry of the Interior and Administration Hospital, Szczecin, Poland; 4 Department of General and Hand Surgery, Pomeranian Medical University, Szczecin, Poland; 5 Department of Radiology, Medical University of Warsaw, Warsaw, Poland; 6 Liver and Internal Medicine Unit, Department of General, Transplant and Liver Surgery, Medical University of Warsaw, ul. Banacha 1a, 02-097 Warsaw, Poland; 7 Translational Medicine Group, Pomeranian Medical University, Szczecin, Poland; 8 Department of Hepatobiliary and Pancreatic Surgery, Churchill Hospital, Oxford University Hospitals NHS Trust, Oxford, UK

**Keywords:** Bile duct injury, Biliary leak, Laparoscopic cholecystectomy, Surgical repair

## Abstract

**Background:**

Iatrogenic bile duct injuries (BDIs) are mostly associated with laparoscopic cholecystectomy but may also occur following gastroduodenal surgery or liver resection. Delayed diagnosis of type of injury with an ongoing biliary leak as well as the management in a non-specialized general surgical units are still the main factors affecting the outcome.

**Case presentation:**

Herein we present three types of BDIs (Bismuth type I, IV and V) following three different types of upper abdominal surgery, ie. Billroth II gastric resection, laparoscopic cholecystectomy and left hepatectomy. All of them were complex injuries with complete bile duct transections necessitating surgical treatment. All were also very difficult to treat mainly because of a delayed diagnosis of type of injury, associated biliary leak and as a consequence severe inflammatory changes within the liver hilum. The treatment was carried out in our specialist hepatobiliary unit and first focused on infection and inflammation control with adequate biliary drainage. This was followed by a delayed surgical repair with the technique which had to be tailored to the type of injury in each case.

**Conclusion:**

We emphasize that staged and individualized treatment strategy is often necessary in case of a delayed diagnosis of complex BDIs presenting with a biliary leak, inflammatory intraabdominal changes and infection. Referral of such patients to expert hepatobiliary centres is crucial for the outcome.

## Background

**Fig. 1:**
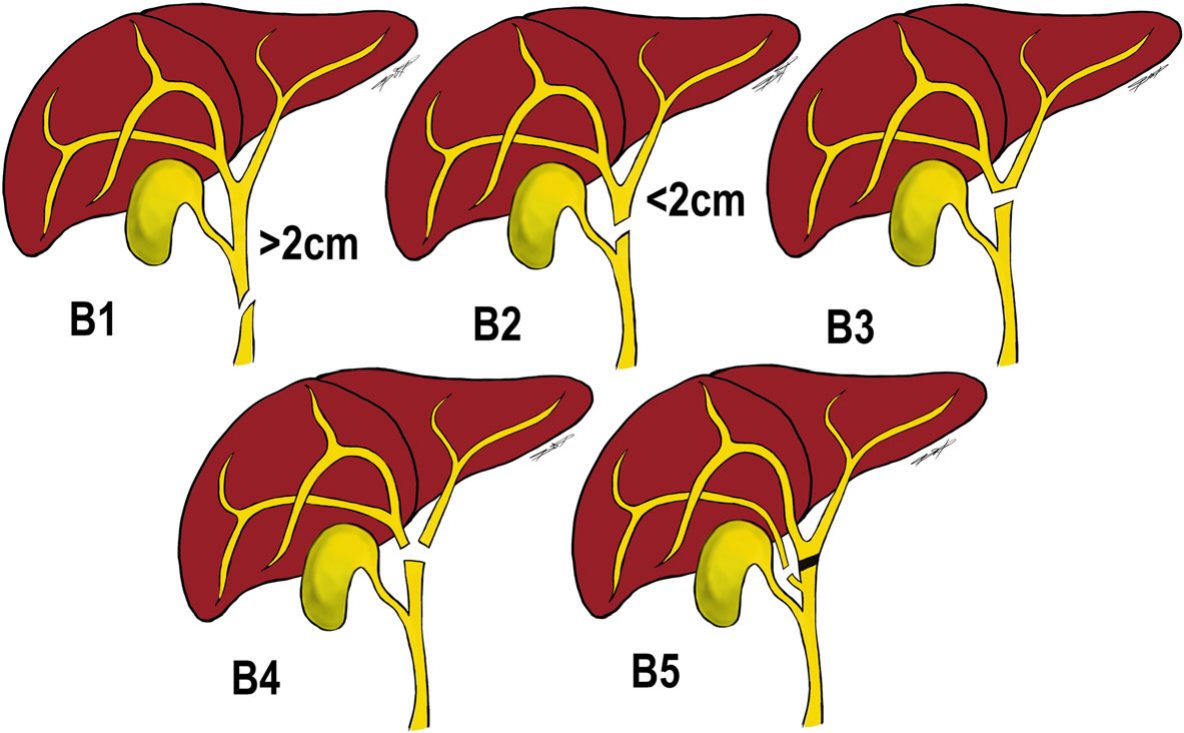
Bismuth classification of Bile Duct Injuries (BDIs) according to the distance of place of injury from the bile duct bifurcation (type 1–4) as well as individual right sectoral bile duct injury (type 5)

Iatrogenic bile duct injuries (BDIs) are potentially life-threatening with high morbidity and mortality. They mainly occur as a complication of laparoscopic cholecystectomy with a constant frequency of 3 to 6 per 1000 cases [1, 2]. Although rarely reported, they may also be associated with gastroduodenal surgery or liver resection. Traditionally, BDIs have been classified using the Bismuth’s classification that is based on the location of injury within the biliary tract (Fig. 1) [3]. It includes five types of BDIs according to the distance from the bile duct bifurcation (type I-IV) as well as individual right sectoral duct injury (type V). Type I is the injury of the common bile duct or common hepatic duct > 2 cm from the hepatic duct confluence while type II involves the proximal common hepatic duct < 2 cm from the confluence. In type III, the injury is located at the confluence with the ceiling of it still being intact whereas type IV involves the destruction of the confluence with the left and right hepatic duct being separated [3].

**Fig. 2:**
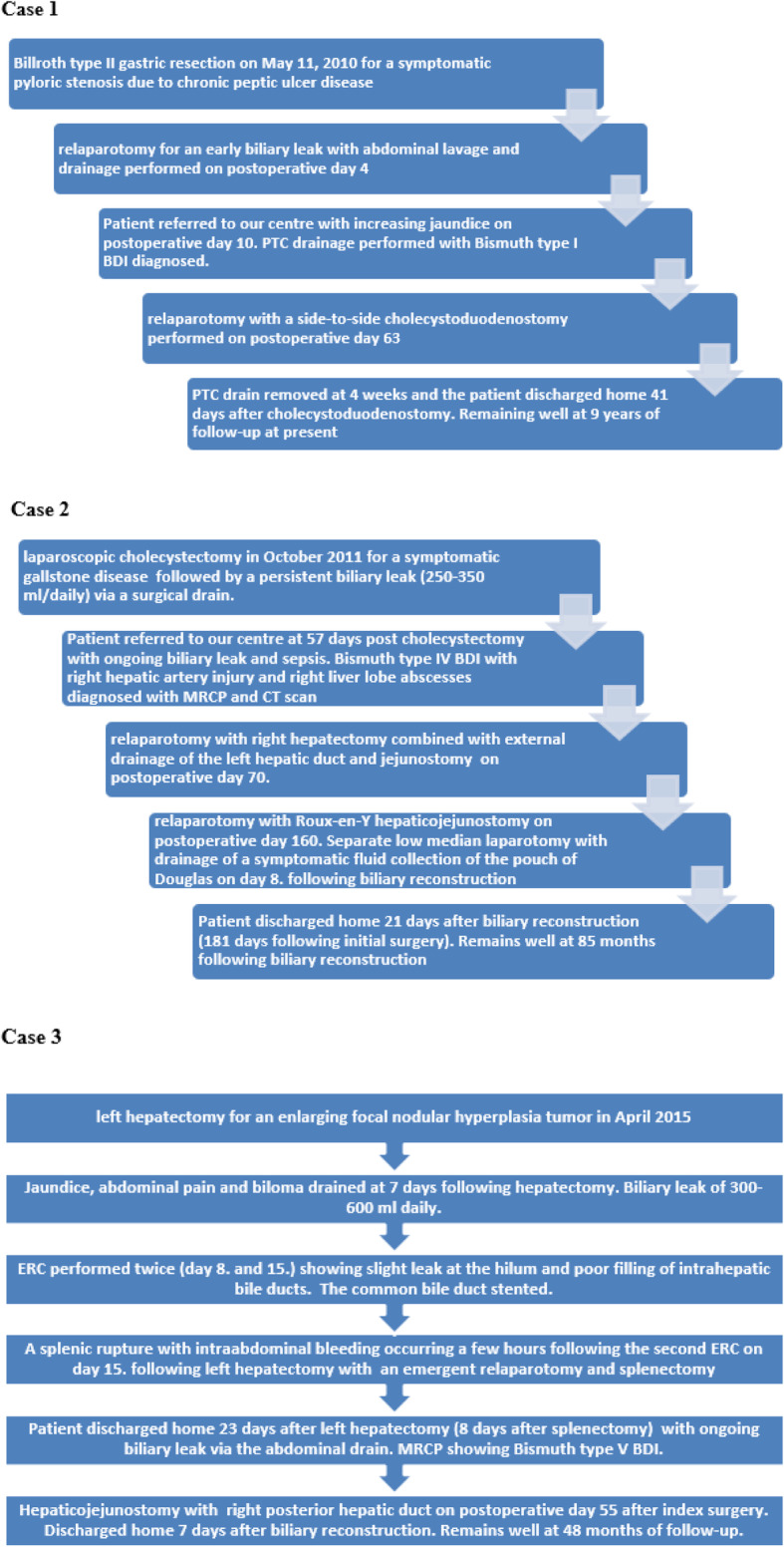
Timeline of complications presenting the sequence of events in the postoperative period of each case

Herein we report a series of 3 patients with complex BDIs sustained during three different surgical procedures, ie. laparoscopic cholecystectomy, Billroth type II gastric resection and left hepatectomy. They differed with regard to the type and mechanism of injury (Bismuth type I, IV and V) [4] with each one requiring an individual treatment strategy (Fig. 2). They were all very difficult to treat mainly because of a delayed diagnosis with a biliary leak resulting in severe inflammatory changes in the right upper abdomen.

## Case presentation

### Case 1

#### Bismuth type I bile duct injury following Billroth II gastric resection

**Fig. 3:**
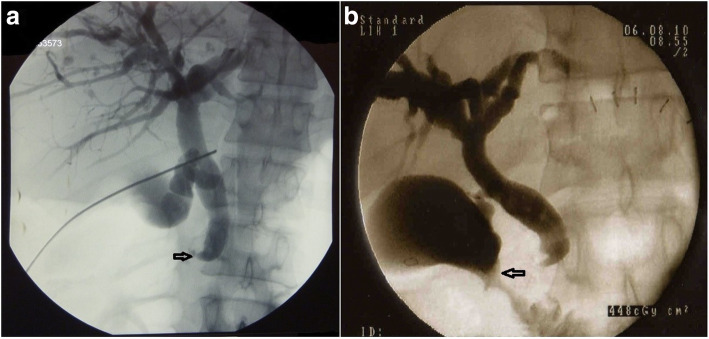
Percutaneous transhepatic cholangiogram (PTC) performed 10 days following Billroth II gastric resection showing a dilated biliary tree and cystic duct with the abrupt stop of the contrast (arrow) within the common bile duct (**a**); The check-up cholangiogram (**b**) performed 3 weeks after cholecystoduodenostomy confirming an undisturbed passage of the common hepatic and common bile duct into the duodenum via an open cystic duct and anastomosis between gallbladder neck and the duodenum (arrow)

A 48-year-old male patient with a symptomatic pyloric stenosis due to chronic peptic ulcer disease underwent Billroth type II gastric resection on May 11, 2010 at an institution elsewhere. The postoperative course was complicated by an early biliary leak, for which a relaparotomy, abdominal lavage and drainage was performed on postoperative day 4. In the following days, the patient presented with signs of jaundice, dilated biliary tree and the gall bladder on abdominal ultrasound and severe wound infection. At this stage the patient was transferred to our institution. A percutaneous transhepatic cholangiography (PTC) drainage was performed (day 10) showing an abrupt ‘cut off’ of contrast at the level of the supraduodenal common bile duct (Fig. 3a). The PTC confirmed a Bismuth type I BDI [3]. Abdominal computed tomography (CT) confirmed that the portal vein and the hepatic artery were patent and uninjured. A PTC drain was left in place for external biliary drainage, together with antibiotic therapy, until clinical and laboratory signs of surgical site infection subsided. All the bile that was drained externally (750–1000 ml/daily) was recirculated back to the gastrointestinal tract together with enteral feeding supplied via a nasojejunal feeding tube.

At relaparotomy (63 days following initial surgery), the signs of severe inflammatory changes within the hepatoduodenal ligament together with peripancreatic tissue necrosis were noted. This precluded both safe dissection of the common hepatic duct and the planned hepaticojejunostomy. In view of a large diameter of the cystic duct (Fig. 3a) and the proximity of the gall bladder neck to the superior surface of the duodenum, a side-to-side cholecystoduodenostomy was created. This proved to be a straightforward procedure that could be performed outside the area of inflammation. The external drainage of bile via a PTC drain was continued for 3 more weeks until a check cholangiogram confirmed a functioning biliary bypass anastomosis (Fig. 3b). The postoperative course was complicated by a prolonged wound infection requiring local drainage and antibiotics. The PTC drain was removed 4 weeks following surgery and the patient was discharged home 41 days after cholecystoduodenostomy (103 days following initial surgery). He remains well at 9 years of follow-up at present.

### Case 2

#### Bismuth type IV bile duct injury with concomitant right hepatic artery injury following laparoscopic cholecystectomy

**Fig. 4:**
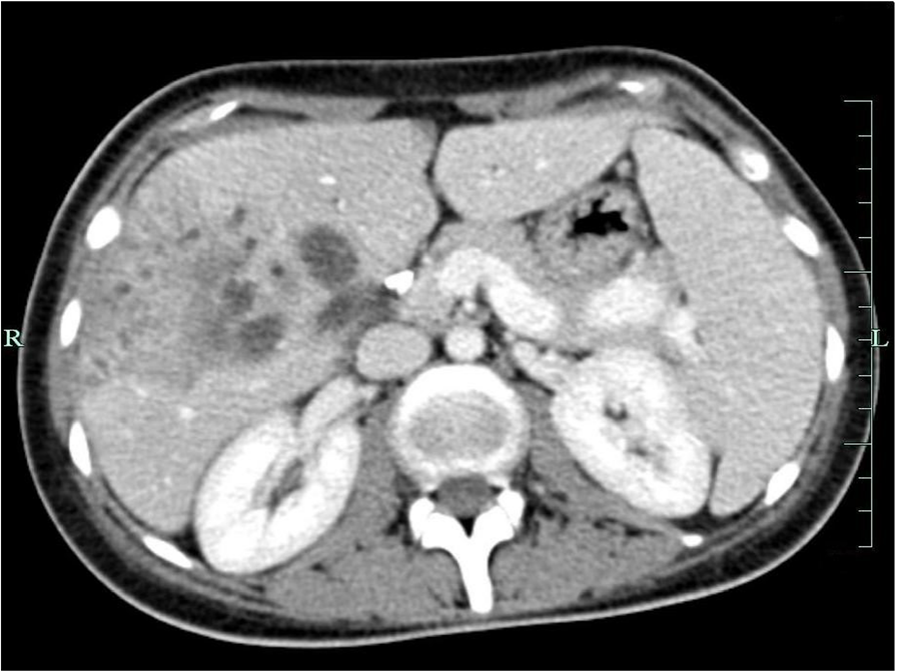
Computed tomography (CT) scan showing right liver lobe ischemia and abscess 2 months following laparoscopic cholecystectomy with combined bile duct and right hepatic artery injury

**Fig. 5:**
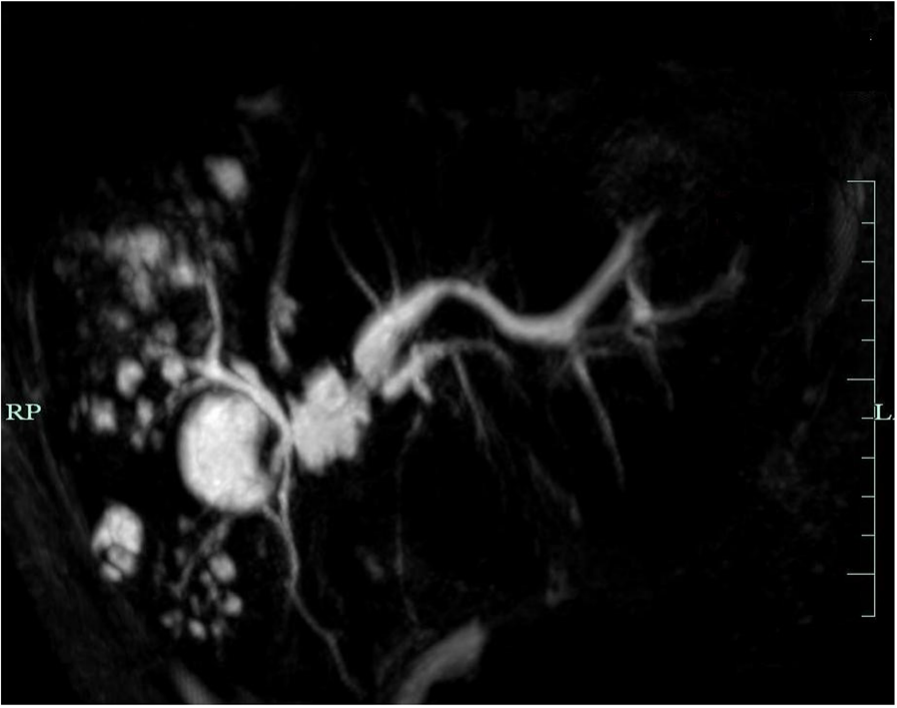
Magnetic cholangiopancreatography (MRCP) showing a bile duct injury high up at the liver hilum with complete separation of the right and left hepatic ducts

A 26-year-old female patient with a symptomatic gallstone disease underwent a laparoscopic cholecystectomy in October 2011 elsewhere. The postoperative course was complicated by a persistent bile leak via a surgical drain (250–350 ml/daily). At 57 days post cholecystectomy the patient was referred to us with an ongoing bile leak and sepsis. On admission, the patient was found jaundiced (serum bilirubin of 5.6 mg/dl) with a bile leak (200–400 ml/daily) and right lobe liver abscesses (Fig. 4) confirmed on CT scan. No blood flow was seen in the right hepatic artery at both Doppler ultrasound and CT angiography. At the same time, a BDI involving the hepatic duct confluence (Bismuth type IV) [3] was demonstrated on magnetic resonance cholangiopancreatography (MRCP) (Fig. 5). Broad spectrum antibiotic therapy was started and an endoscopic, transgastric ultrasound-guided left hepatic duct cholangiogram revealed obstructive jaundice of the left liver lobe, for which a transgastric drainage (8.5 Fr) was done as the first line therapy. This resulted in control of infection and improvement of general condition of the patient, who was then scheduled for a re-laparotomy with right hepatectomy. During re-laparotomy (day 70. following the index surgery), we found several right lobe ischemic abscesses and severe inflammatory changes within the liver hilum. A right hepatectomy combined with copious abdominal lavage was performed. Additionally, a straight 12 F Silastic drain was inserted into the left hepatic duct and secured with a single nonabsorbable polypropylene 4/0 suture to the bile duct wall and delivered externally. We also placed a feeding jejunostomy tube in the proximal jejunum in order to recirculate bile back into the bowel, in preparation for future biliary reconstruction as a staged procedure. The postoperative course was uneventful. The patient was instructed how recirculate bile drained externally back after meals via the jejunostomy. She was discharged home with two abdominal (biliary and jejunostomy) drains, 15 days following right hepatectomy. The patient was then seen in the surgical outpatient department every 2–3 weeks before being finally admitted for definitive biliary reconstruction.

At relaparotomy, performed 160 days following initial surgery (90 days after right hepatectomy), multiple dense upper abdominal adhesions were found and divided. Once this was done, nearly complete resolution of all the inflammatory changes within the liver hilum was noted and the left hepatic duct identified along the external biliary drain that had initially been placed inside its lumen. There was a significant anti clock-wise rotation of the compensatively hypertrophic left liver lobe making the access to the duct challenging. The stump of the left duct to be anastomosed had been displaced deep down dorsally making the posterior part of the biliary anastomosis of Roux-en-Y hepaticojejunostomy very difficult to perform. The postoperative course was complicated by a low volume bile leak (100–150 ml/daily) which continued for nearly 2 weeks before it stopped. In the meantime, a symptomatic fluid collection located in the pouch of Douglas was drained surgically (day 8.) via a separate, low median laparotomy. The patient was discharged home 21 days after biliary reconstruction (181 days following the initial surgery). She remains well at present at 90 months post cholecystectomy and 85 months following biliary reconstruction.

### Case 3

#### Bismuth type V bile duct injury following left hepatectomy

A 31-year-old female patient underwent left hepatectomy for an enlarging focal nodular hyperplasia tumor in April 2015. The diagnosis was confirmed by magnetic resonance imaging, which clearly showed typical features of focal nodular hyperplasia. Nevertheless, the patient was accepted for surgery due to the significant growth of the tumor (up to 6 cm in diameter) which doubled its size within 2 years. Following surgery, the patient presented with increasing jaundice (bilirubin of 9 mg/dl on day 7.) and abdominal pain with vomiting. Abdominal ultrasound confirmed a large fluid collection that was adjacent to the liver transection plane and proved to be bilious in origin at percutaneous drainage. A drain was left inside the collection and a biliary leak of around 300–600 ml/daily continued. At endoscopic retrograde cholangiography (ERC) which was performed twice, a slight leak combined with poor filling of the intrahepatic bile ducts was shown. At that time we believed the compromised filling of the intrahepatic bile ducts was due to the narrowing at the confluence. The common bile duct was stented endoscopically with a plastic (8.5 Fr) stent (day 8.) and as the leak continued, a self-expandable metallic stent (day 15.) was inserted. A few hours following the second ERC procedure, the patient presented with sudden abdominal pain, hypotension and a drop in hemoglobin from 12.2 to 6.6 mg/l due to a splenic rupture that was confirmed by the CT scan. An emergent relaparotomy was performed. During surgery, the patient required blood transfusion with inotropic support and her condition was unstable. In view of this, surgery was limited to splenectomy, abdominal lavage and drainage. Considering dense adhesions within the hepatoduodenal ligament and no obvious source of a biliary leak visible, no intraoperative investigations of the biliary tree were pursued. Following splenectomy the condition of the patient improved, however the biliary leak persisted. At this stage, it was thought to originate from the cut surface of the liver. Therefore, the patient was discharged home (day 23. and 8. following left hepatectomy and splenectomy, respectively) with the abdominal drain in-situ for further management in the outpatient clinic.

**Fig. 6:**
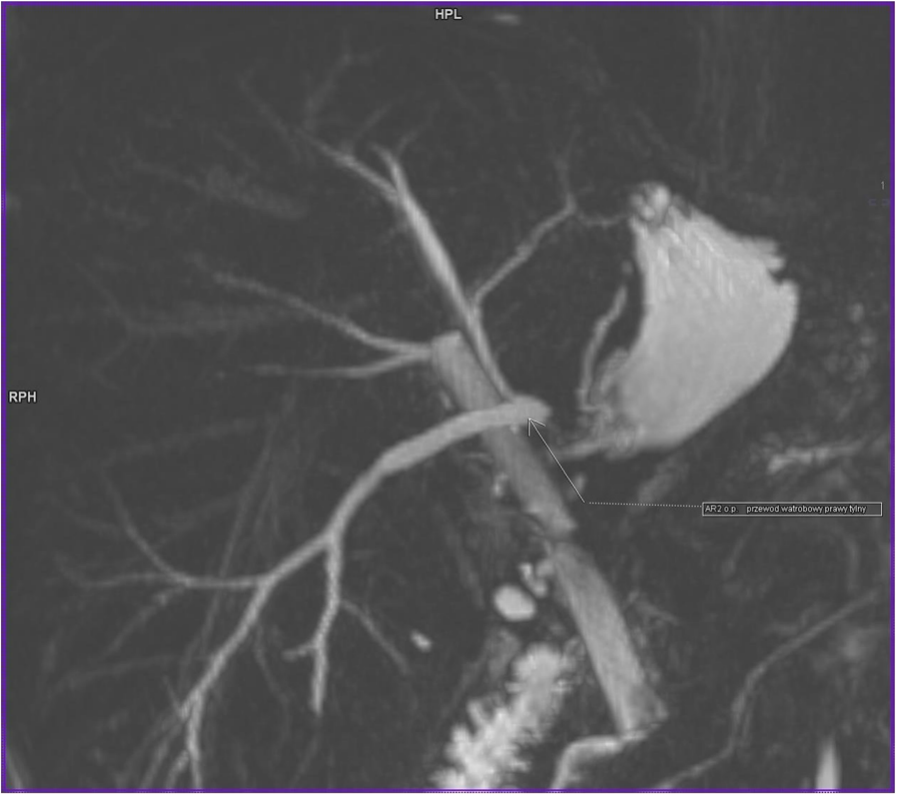
Magnetic cholangiopancreatography (MRCP) showing a transected right posterior sectoral duct about 6 weeks following left hepatectomy (arrow). The self-expandable metallic stent in the common bile duct is also visible

**Fig. 7:**
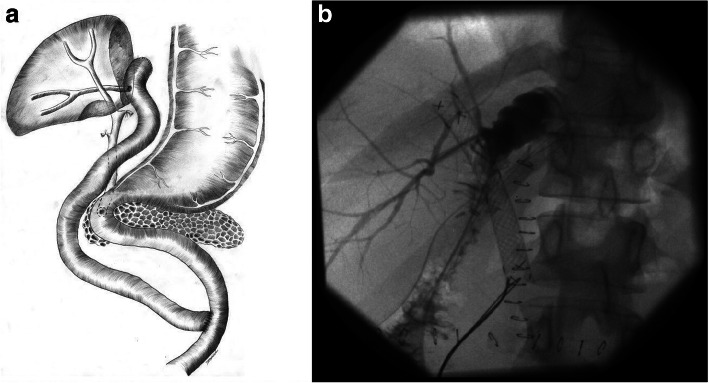
Roux-en-Y hepaticojejunostomy (schematic) with the aberrant right posterior sectoral duct (**a**). The check-up transanastomotic bile drain cholangiogram (**b**) showing an open hepaticojejunostomy with the contrast flowing from the right posterior sectoral duct into the Roux limb of jejunum. The self-expandable metallic stent in the common bile duct is also clearly visible

As the biliary leak continued, MRCP was carried out. This revealed a Bismuth type V [3] BDI with a leak from the right posterior sectoral duct that had no communication with the remaining biliary tree being previously stented with the metallic stent (Fig. 6). The patient was then offered surgical repair, which was performed 55 days following initial surgery. During relaparotomy, an open stump of the right posterior sectoral duct measuring around 3 mm of diameter was identified at the transection plane close to the liver hilum. A Roux-en-Y hepaticojejunostomy over a transanastomotic external biliary drain was created (Fig. 7). The postoperative course was uneventful and the patient was discharged home 7 days after surgery. The self-expandable metallic stent was endoscopically removed at 7 months following primary surgery. She remains well and asymptomatic at 48 months of follow-up with undilated biliary tree on ultrasound and normal values of liver function tests.

## Discussion and conclusions

Iatrogenic bile duct injuries (BDIs) are mostly associated with laparoscopic cholecystectomy but may also occur following gastroduodenal surgery or liver resection [1, 2]. Herein, we described three BDIs of different types (Bismuth I, IV, V), which occurred following three different upper abdominal surgical procedures and were very difficult to manage. Complex BDIs represent complete bile duct transections with or without loss of biliary tissue. In such cases, typically classified according to the Bismuth-Strasberg classification (Fig. 1) [3, 4], no endoscopic and/or radiological therapy is feasible and the patients require surgical remedies [5]. These patients must be managed in tertiary hepatobiliary (HPB) centres where surgical expertise and experience in managing such injuries exist. Repair of BDI are at times also possible during the index surgery. However a majority of BDIs; approximately 75–80%, are not recognized at the time of cholecystectomy [6–8]. A specialist hepatobiliary surgeon is also usually not available on call for an immediate on-table repair of the injury. Therefore, early transfer of patients to a specialized HPB unit once the diagnosis of a BDI is recommended [9–12]. This has been shown to improve outcome, reduce morbidity, duration of hospital stay and costs [9–13]. A delayed diagnosis of BDI resulted in very complicated postoperative course, long hospital stay and poor quality of life for a long period of time in each of our patients. All efforts should therefore be made to diagnose and manage a biliary complication early by an experienced hepatobiliary team.

No clear-cut guidelines exist to define the timing of biliary repair. However, active intraabdominal infection or sepsis, biliary peritonitis and/or concomitant vascular injury are all associated with much worse outcome if an early primary repair is attempted [14, 15]. This clearly supports the practice of sepsis control and/or biliary drainage as the first line of treatment. Two of our patients were referred to us with signs of sepsis and biliary peritonitis as a result of a delayed BDI recognition. Considering this, they were both not suitable for an immediate repair. The first patient sustained an unrecognized, Bismuth type I common bile duct injury (Fig. 3a) during a Billroth II gastric resection. Despite the presence of a biliary leak confirmed during relaparotomy 4 days following gastric resection, no intraoperative diagnostics of the biliary tree continuity were pursued for unknown reasons. Once the intraoperative cholangiogram had been done, the type of BDI could have been recognized and managed without further delay. In difficult cases of unclear bile duct anatomy with anticipated risk of injury, intraoperative use of fluorescent or transcystic cholangiogram is indicated in order to delineate biliary anatomy and prevent BDIs. Fluorescent cholangiogram that is used in some centres as an alternative to classic transcystic cholangiogram involves intravenous administration of indocyanine green prior to surgery. This is excreted into the biliary system and allows the surgeon to identify the biliary tree by reflecting infrared light prior to any major dissection or cutting [16]. In such cases, if the injury is recognized during surgery, a choledochoduodenostomy or a hepaticoduodenostomy may be considered [5, 17]. However, choledocho- or hepaticoduodenostomy is associated with a high incidence of recurrent cholangitis following surgery and a substantial risk of a fatal outcome if the anastomosis breaks down [18]. Therefore, the use of a Roux limb is always preferable if the bile duct has been transected completely. This is mainly because the communication between the pancreaticoduodenal, gastroduodenal and the right hepatic arteries is always disrupted in such cases. This results in the distal arteries of the bile duct becoming the end arteries. Such arteries provide the most tenuous blood supply in the distal portion of the bile duct as compared to the rich supply high up at the bile duct confluence [19, 20]. Therefore, biliary-enteric reconstruction performed as close to the biliary confluence as possible provides the best chance for a good outcome. However, this was not feasible in our patient due to persisting, severe inflammatory changes around the hepatoduodenal ligament. Considering a wide diameter of the cystic duct (Fig. 3a) and no inflammatory changes within the gall bladder and duodenal wall, we decided to create an anastomosis between the gall bladder neck and the duodenum (Fig. 3b) [21], which in the long-term proved to be a successful and durable option.

BDIs during laparoscopic cholecystectomy may often occur due to the lack of culture of safety with no critical view of safety achieved prior to clipping or division of any biliary structures. The universal use of critical view of safety has been shown to be effective in preventing both BDIs and asscociated hepatic artery injury at cholecystectomy [22]. This consists of cleaning of the hepatocystic triangle from fat and connective tissue demonstrating 2 and only 2 tubular structures (i.e. the cystic duct and the artery) entering the gallbladder. When this cannot be safely achieved, the operator should opt for adjunctive intraoperative imaging or other bailout strategies [22]. BDIs with loss of hepatic duct confluence following laparoscopic cholecystectomy, classified as Bismuth type IV may also result from an anatomical variation in which a low extrahepatic biliary confluence is present. These are often accompanied by the right hepatic arterial injuries [9, 23] which was also seen in the second of our patients in whom the viability of biliary tree and parenchyma of the right hemi-liver became seriously compromised (Fig. 4). This was due to the confluence disruption (Fig. 5), which impaired the development of collateral circulation within the hilar plate [24]. By contrast, when the biliary confluence is preserved, outcome following repair at that level have good outcomes, irrespective of associated arterial injury [23, 24]. This is due to a rich vascular network at the confluence that allows a compensatory supply from the left to the right side of the biliary tree. We have previously shown that a wide biliary-enteric anastomosis with the hepatic duct confluence extended by the anterior opening of the left duct (Hepp-Couinaud technique) provides a good outcome for Bismuth type I-III injuries [25]. In case of Bismuth type IV injuries, therapeutic options include various types of biliary-enteric anastomosis with creation of neo-confluence, double-barrel (separate right and left) anastomosis, the use of metallic stents as well as a major hepatectomy or even liver transplantation [26, 27]. Hepatic resection is considered necessary if a vascular injury results in severe devascularization of part of the liver or if a major injury to the hepatic duct cannot be drained effectively by a biliary-enteric anastomosis. Moreover, arterial injury may be considered an extra indication to delay biliary repair given concern for biliary or biliary-enteric anastomotic stricture [14, 24]. This was the case in the second of our patients. Liver transplantation may be indicated in extreme cases with a combined vascular and biliary injury resulting in acute liver failure or if secondary biliary cirrhosis develops [26, 28].

As already mentioned, if sepsis and biliary peritonitis predominate, a percutaneous or surgical drainage is necessary as the first line treatment [14, 15]. This was provided in the first two cases giving a good long-term outcome. The external drainage of the left duct for several weeks in the second patient (following right hepatectomy) resulted in nearly complete resolution of all of the inflammatory changes within the liver hilum and the left duct. Therefore, we were able to perform a biliary-enteric reconstruction in a non-inflamed operative field. The problem we encountered during surgery in this patient was a significant, anti-clock wise rotation of the remnant left liver lobe into the right subphrenic space. This made the back wall of hepaticojejunostomy very difficult to perform, resulting most likely in suboptimal suture placement and a biliary leak. Hepatopexy, i.e. suturing of the divided falciform ligament to the abdominal wall following right hepatectomy may prevent or at least diminish the risk of left liver lobe rotation and related to it complications [29]. Additionally, performance of the anastomosis over a transanastomotic external biliary drain may have reduced the risk of a biliary leak that occurred in the postoperative period in our patient. This possibly might have prevented the development of a fluid collection in the pouch of Douglas that required another surgery later on.

**Fig. 8:**
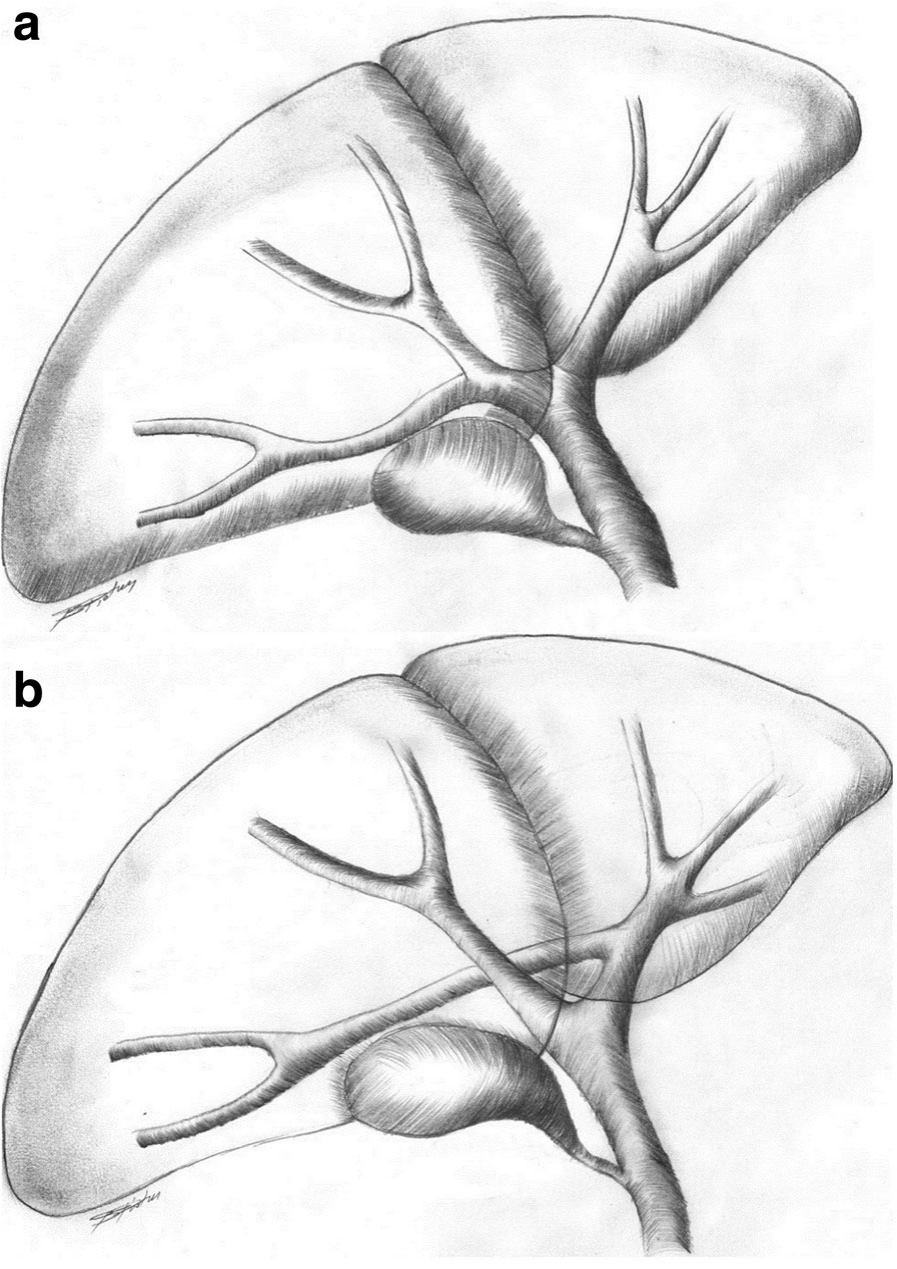
Typical hepatic duct confluence (**a**) with common hepatic duct bifurcation into the right and left hepatic ducts. A dangerous anatomical variant of right posterior sectoral duct (**b**) draining into the left hepatic duct at a distance from the confluence

Biliary complications may also occur following liver resection. However, these have been rarely reported being probably often classified as biliary leaks from the transection plane. Some of them may result from anatomic variations within the biliary tree [30] with aberrant right hepatic duct being the most common. We have previously described an isolated right posterior BDI sustained during laparoscopic cholecystectomy, when misidentification of the duct draining into the gall bladder, the cystic or common hepatic duct may cause the biliary problem [31]. The right posterior duct may also originate from the left hepatic duct in about 6–16% of individuals [32], (Fig. 8). In such cases, the right posterior sectoral duct is at risk of an injury while left hepatectomy is being done. Prevention of this complication includes division of liver parenchyma as far as possible towards the left from the common hepatic duct bifurcation. If this is not possible (like in tumors located close to the liver hilum), MRCP prior to left hepatectomy may alert the surgeon on biliary anatomical variants at risk for injury. Such injury occurred in patient number three in this series in whom a seemingly “straightforward” left hepatectomy resulted in a cascade of complications later on. This started with a significant delay in proper diagnosis of type of BDI, which was only identified by MRCP (as Bismuth type V) 4 weeks following left hepatectomy (Fig. 6). Had we done it once the diagnosis of a biliary leak was made (day 7.), immediate relaparotomy with hepaticojejunostomy may have been possible. This would have also probably made the second ERC procedure unnecessary, avoiding emergent relaparotomy for splenic rupture that occurred following ERC. Among possible mechanisms responsible for the spleen rupture following upper endoscopic procedures, either direct trauma or tearing of splenic capsule due to excessive pulling on the splenic ligaments while maneuvering with the endoscope, are considered. This is supported by the reports on splenic ruptures following vomiting or even coughing [33].

In summary, a detailed knowledge and awareness of all possible anatomical variants of biliary tree is crucial for surgeons to avoid complications. Once they occur, the timing of their recognition and a precise diagnosis of type of injury are both essential to plan the treatment properly. In case a delayed diagnosis with significant inflammatory changes within the liver hilum are found, control of infection and adequate biliary drainage becomes priority before a final biliary reconstruction can be performed. Roux-en-Y hepaticojejunostomy remains the reference treatment. However, liver resection or unconventional techniques (like cholecystoduodenostomy) may still be applied in some exceptional cases of BDIs. Referral of such patients to expert HPB centres is crucial for the outcome.

## Data Availability

The data used and analyzed during the present study are completely available in the article and any other can be obtained from the corresponding Author on reasonable request.

## Abbreviations

BDI: Iatrogenic bile duct injury
CT: Computed tomography
ERC: Endoscopic retrograde cholangiography
HPB: Hepatobiliary
MRCP: Magnetic resonance cholangiopancreatography
PTC: Percutaneous transhepatic cholangiography
